# **Antibodies** Against Anthrax: Mechanisms of Action and Clinical Applications 

**DOI:** 10.3390/toxins3111433

**Published:** 2011-11-16

**Authors:** Jeffrey W. Froude, Philippe Thullier, Thibaut Pelat

**Affiliations:** 1 Unité de biotechnologie des anticorps et des toxines, Département de microbiologie, Institut de Recherche Biomédicale des Armées (IRBA-CRSSA), 38702 La Tronche Cedex, France;Email: jeffrey.froude@us.army.mil (J.W.F.); t.pelat@orange.fr (T.P.); 2 US Army Medical Research and Materiel Command, Fort Detrick, MD 21702, USA

**Keywords:** anthrax toxins, protective antigen, lethal factor, edema factor, antibodies

## Abstract

*B. anthracis* is a bioweapon of primary importance and its pathogenicity depends on its lethal and edema toxins, which belong to the A-B model of bacterial toxins, and on its capsule. These toxins are secreted early in the course of the anthrax disease and for this reason antibiotics must be administered early, in addition to other limitations. Antibodies (Abs) may however neutralize those toxins and target this capsule to improve anthrax treatment, and many Abs have been developed in that perspective. These Abs act at various steps of the cell intoxication and their mechanisms of action are detailed in the present review, presented in correlation with structural and functional data. The potential for clinical application is discussed for Abs targeting each step of entry, with four of these molecules already advancing to clinical trials. Paradoxically, certain Abs may also enhance the lethal toxin activity and this aspect will also be presented. The unique paradigm of Abs neutralizing anthrax toxins thus exemplifies how they may act to neutralize A-B toxins and, more generally, be active against infectious diseases.

## 1. Introduction

Anthrax is caused by the gram-negative, spore forming bacterium *Bacillus anthracis*. This lethal disease is still endemic in some parts of the world, primarily to herbivores in the less-developed countries but can affect a wide range of species, including humans. Anthrax is considered a biological threat due to the historical weaponization of this agent since World War II, in addition to recent events, particularly the intentional anthrax letter attacks in 2001 in the United States. *B. anthracis* is classified by the Centers for Disease Control and Prevention (CDC) as a category A select agent, representing the biological agents most at risk of being weaponized [1]. *B. anthracis* pathogenesis depends on three virulence factors, the production of a protective capsule [2] and of two A-B toxins [3]. The A-B (or “binary”) bacterial toxins consist of a two component complex whose “B” subunit is responsible for cell surface binding, and the “A” subunit which is responsible for the enzymatic activity of the toxin [4]. The anthrax toxins are composed of three different proteins, a single receptor-binding B-subunit, designated as protective antigen (PA), and two alternative A-subunits, the lethal factor (LF) and the edema factor (EF). LF interacts with PA to form the lethal toxin (LT) and EF interacts with PA to form the edema toxin (ET) [5].

**Figure 1:**
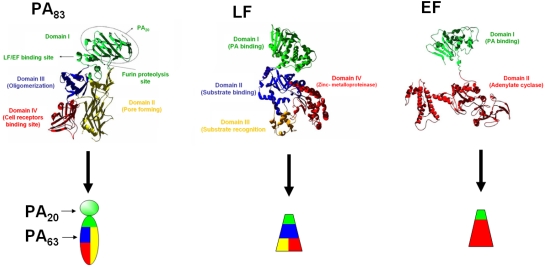
Structures of protective antigen (PA), lethal factor (LF) and edema factor (EF) subunits. PA structure has been obtained using file 1acc from the Protein Data Bank [6]. LF and EF structures are derived from the files 1j7n [7] and 1xfv [8], respectively. For each subunit, the different domains are identified on ribbon models and their respective function is indicated. The color code utilized on ribbon models was re-utilized for schematic rendering.

The crystal structure of PA_83_ has been resolved [6] (Figure 1) and shows four different domains, each playing a different role in the intoxication mechanism (residues in this review are designated according to their numbering in three-dimensional structures). Domain I (residues 1-258) contains the furin proteolysis site [6], and the LF/EF binding site. Domain II (residues 259-487) is involved in heptamer and pore formation, and interacts with anthrax toxin receptors (ATRs) [9,10]. Domain III (residues 488-595) is also involved in heptamer formation [11]. Domain IV (residues 596-735) is essential in the recognition and binding to the cellular ATRs [12,13]. 

**Figure 2:**
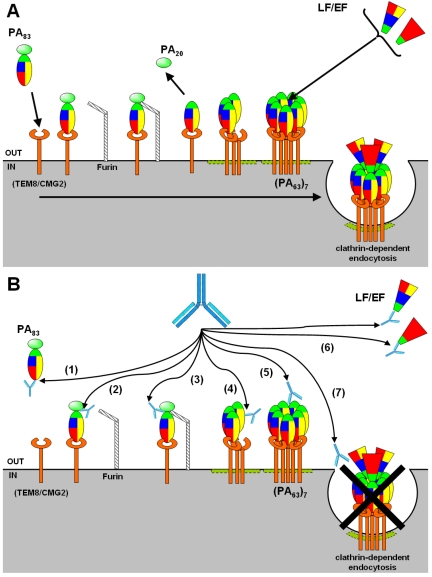
The different steps of anthrax toxins entry, and their inhibition by antibodies. (**A**) Various steps of anthrax toxins entry. PA_83_ binds to its cell receptors and is processed by furin on the cell surface. PA_20_ is released and PA_63_ remains attached to the receptor. Heptamerization of PA_63_ induces the formation of LF/EF binding site. The toxin complex is then endocytosed. (**B**) Inhibition of the various steps of anthrax toxins entry by Abs. Neutralizing Abs act at each entry step: binding of PA_83_ to its receptors (1), PA_83_ cleavage by furin (2), PA_20_ release (3), PA_63_ heptamerization (4), LF/EF binding to the heptamer by targeting PA (5) or LF/EF (6), and endocytosis of the toxin (7).

Toxin entry into host cells involves several steps. First, PA in the form of an 83-kDa protein (PA_83_) binds to ATRs, the tumor endothelial marker 8 (TEM-8) and the capillary morphogenesis protein-2 (CMG-2) [14,15]. PA_83_ amino-terminal 20-kDa region (PA_20_, residues 1-167) is then proteolytically cleaved by a furin-like protease and released (Figure 2). The PA_63_ fragment remains bound on cell surface and forms a homo-heptameric structure that binds EF or LF, and promotes their cell entry by a clathrin-dependant endocytosis. LF is a zinc-dependent protease specific for the mitogen-activated protein kinase kinase family [16,17] and EF is a calmodulin-activated adenylyl cyclase [8,18].

The crystallographic structure of LF has also been resolved (Figure 1) [7]. LF is composed of four different domains. Domain I (LF_N_; residues 1-254) interacts with PA [19]. Domain II (residues 263-297 and 385-550) presents a pocket which captures its proteolysis substrate. Domain III (residues 303-382), inserted within domain II, plays a role in the enzymatic specificity [7,20]. Domain IV (residues 552-776) contains the catalytic center (HExxH), where the first H is localized at position 686, E is localized at 735 and the last H is at position 690.

The EF structure was the last to be resolved (Figure 1) [8] and it presents two functional domains, domain I (EF_N_, residues 1-291) and domain II (residues 292-798). EF_N_ interacts with PA in the same way as the LF_N_. Domain II is responsible for the adenylate cyclase activity of EF, leading to an uncontrolled increase in intracellular cAMP concentration in host cells [8,18]. 

Antibodies (Abs) against anthrax have historically demonstrated therapeutic potential and, in their recombinant form, represent several products currently advancing into clinical trials. Indeed, the first specific treatment of anthrax, Achille Sclavo’s serum, consisted of polyclonal antibodies. This bovine derived product, used in the 1890’s, increased survival rates of cutaneous anthrax from 75 to 96% [21]. Antibiotics have now replaced the Abs as main molecules for anthrax treatment but they have limitations which, in turn, may be compensated by Abs. In effect, current antimicrobials (fluoroquinolone, tetracycline or penicillin G) [22,23] require early administration in order to provide protection, due to the rapid course of infection and toxins secretion after exposure to *B. anthracis* spores by the pulmonary route. Additionally, this treatment must be continued for 60 days. Following the 2001 anthrax letter attacks, the compliance for this treatment was only about 40% [24,25]. Abs targeting anthrax toxins could be used to reduce this lengthy treatment time. The natural emergence of antimicrobial resistant strains [26,27], as well as resistant strains of anthrax obtained *in vitro*, demonstrates the need for alternate treatments [28,30]. Consequently, Abs are generally expected to increase the therapeutic window, decrease the length of treatment, and overcome potential antibiotic resistant strains. Abs enabling these functions, by targeting the pathogenic mechanisms at various steps involving LT/ET toxicity, will be reviewed in this article. The current field of Abs against anthrax includes multiple anti-PA Abs under clinical testing as well as several anti-LF, anti-EF, and anti-capsule Abs. Additionally the paradoxical role of Abs, enhancing toxicity, will be briefly discussed. Of note, the scope of the manuscript is not to correlate Abs and vaccination, but rather how monoclonal and recombinant Abs act.

## 2. Antibodies Neutralizing the Anthrax Toxins

PA has been regarded as the most important target of neutralizing Abs because (i) it plays a central role for the formation of both LT and ET, (ii) it is involved at the earliest stages of the intoxication process, and (iii) vaccines composed of PA showed that PA-binding Abs effectively limit *B. anthracis* pathogenicity. Several anti-PA monoclonal antibodies (mAbs) have been isolated and in certain cases, their neutralization mechanisms were studied. Interestingly, neutralizing Abs directed against the four domains of PA were described, and neutralized at each step involving this subunit: PA interaction with ATRs, PA proteolysis by furin and the release of PA_20_, PA heptamerization, PA interaction with LF/EF, and the endocytosis of the toxin complex (Figure 2). As an alternative to anti-PA, Abs against LF and EF have been considered. These Abs act at the latest stages of the toxin entry: the interaction of LF/EF with the heptamer and the endocytosis of LT/ET. The protective efficacy of anti-LF Abs has been shown in *in vivo* models of the disease, often with a better efficacy than anti-PA [31].

### 2.1. Antibodies Inhibiting PA/Receptors Interaction

The first anthrax toxin entry step involves the high affinity (*K*_D_ = 170 pM) binding of PA to one of the two known ATRs [32] (Figure 2). Only the extracellular domain of these receptors, related to the von Willebrand factor A (vWFA) domain [33], is involved in the anthrax toxin entry. The vWFA domains share 60% of their amino acids, and include a metal ion-dependent adhesion site (MIDAS) motif which is most likely involved in their interaction with PA. Mutagenesis studies have shown that the carboxylate group of aspartate, at position 683 on PA, plays a critical role in the PA-receptors interaction by completing the coordination of MIDAS [34]. The X-ray crystal structure of PA complexed with the vWFA domain of CMG2 has been resolved and identified the four PA loops that are essential for ATRs binding: the loop between residues 340-348 (domain II), between residues 654-662, 681-688, and 714-716 (domain IV) [9].

**Figure 3:**
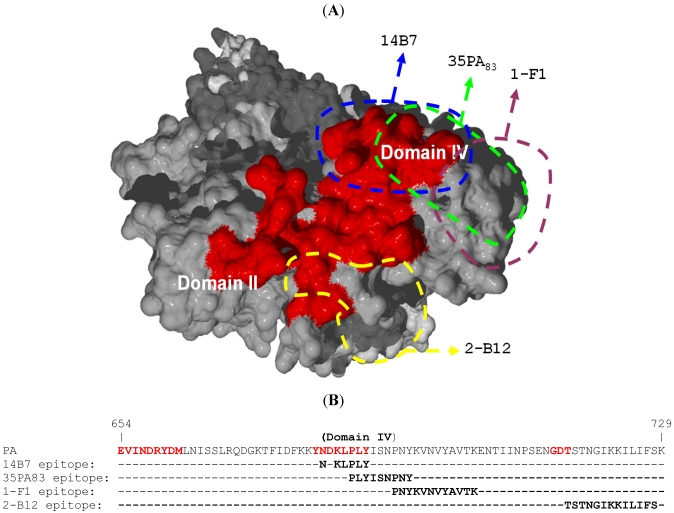
Neutralizing epitopes inhibiting PA-ATRs interactions compared with anthrax toxin receptors (ATRs) binding site. (**A**) Three-dimensional localization of the neutralizing epitopes on PA. PA structure has been obtained using file 1acc from the Protein Data Bank [6] and was colored in grey. Residues of the four PA loops constituting the ATRs binding site, residues 340-348 (part of domain II), residues 654-662, 681-688, and 714-716 (part of domain IV) were colored in red. The core of the four well-defined PA neutralizing epitopes were delimited in blue for 14B7, in green for 35PA_83_, in purple for 1-F1 and in yellow for 2-B12. After Ab binding to the core epitope, the interface between Ab and PA should cover a larger surface. (**B**) Localization of the neutralizing epitopes on PA sequence. Localization of the four epitopes was given as sequence alignments; residues constituting ATRs binding site were colored in red on PA sequence.

The first mAbs, whose neutralization properties were tested *in vitro* and *in vivo*, inhibited the interaction between PA and the vWFA domains of the ATRs. In particular, 14B7, an Ab with a high affinity (*K*_D_ = 0.33 nM), neutralized LT and ET by blocking PA interaction with cells [35]. In a study of 36 anti-PA mAbs, only two were neutralizing: 3B6 and 14B7. Competitive binding experiments have shown that 3B6 and 14B7 recognized the same epitope on PA. Utilizing a mutagenesis strategy, the precise epitope of 14B7 was identified as residues 682, 684, 685, 686, 687 and 688 [13] (see sequence alignment on Figure 3). This neutralizing epitope corresponded to one of the four important loops on PA, involved in its interaction with ATRs, thus explaining the high efficiently of 14B7. This loop was additionally targeted by other neutralizing Abs such as 35PA_83_, a macaque-derived Ab of high affinity (*K*_D_ = 3.4 nM) which neutralized LT *in vitro* (IC_50_ = 5.6 nM) [36], and whose epitope have been localized between residues 686 and 694 by Pepscan analysis. With the same technology, murine mAbs 1-F1 and 2-B12 epitopes were precisely localized between residues 692-703 and 716-727 respectively, two regions that are immediately adjacent to the 14B7 binding site [37]. The epitope of two chimpanzee Abs, W1 and W2, which prevented the binding of PA to RAW265.7 cells, were localized between residues 614-735 and this region encompassed three of the four PA loops involved in the ATRs interactions [38]. Identically, Mab 7.5 bound to PA domain IV between residues 608 and 735 [39]. Several anti-PA Abs, directed against domain IV of PA, act by inhibiting PA interaction with its cell receptors but their epitopes were not precisely mapped [31,40,41,42]. These Abs include Raxibacumab, a human IgG1, also designated Abthrax^TM^, isolated using phage display technology [43].

Among the Abs presented above, two have entered clinical trials for therapeutic approval, Raxibacumab and Anthim^®^, derived from 14B7. Raxibacumab was shown to be protective both prophylacticly and therapeutically in a non-human primate (NHP) model using a 40 mg/kg intravenous (i.v.) dose, eliciting respective protections of 90% (100 LD_50_) and 64% (200 LD_50_) against aerosolized Ames spores. Tolerance data was evaluated in a group of human volunteers [44] and a total of 65,000 doses of Raxibacumab has been ordered for the US Strategic Stockpile while this mAb awaits final FDA approval [41]. Anthim^®^ (Elusys, Pine Brook, NJ), previously ETI-204, was developed from the murine mAb 14B7 as a chimeric deimmunized mAb utilizing the DeImmunisation^®^ process (Antitope, Cambridge, UK) [45]. *In vivo* analysis utilizing the rabbit Ames spore inhalation model demonstrated complete prophylactic (163 LD_50_) and 80% therapeutic (172 LD_50_) protection when ~4 mg/kg of Anthim^®^ was administered by i.v. Although the results have not been published, Anthim^®^ has completed Phase I clinical trials both alone and in combination with antibiotics under National Clinical Trials (NTC) study designations NCT00829582 and NCT00138411.

### 2.2. Antibodies Inhibiting PA Cleavage by Furin

After PA_83_ has bound ATRs, it is cleaved by a cell membrane-associated furin into two proteins, a 63 kDa (PA_63_) and a 20 kDa (PA_20_) fragment, with PA_63_ remaining bound to the receptor [46]. This cleavage occurs at R167, part of the RKKR cleavage site (residues 164-167), which is localized in an exposed loop of the domain I [6]. This cleavage is necessary to expose a large hydrophobic surface on PA_63_ which binds either LF or EF [47,48]. 

Murine mAb 48.3 [39] is an Ab protective *in vivo* against *B. anthracis*, which was shown to block this enzymatic cleavage in an assay where PA_83_ and mAb 48.3 were incubated on CHO-K1 cells. The authors report that, when the concentration of mAb 48.3 increased, the abundance of PA_63_ decreased thus showing inhibition of PA_83_ cleavage. The epitope of mAb 48.3 was localized between residues 314-608, according to the reactivity of mAb 48.3 with various PA fragments. This epitope was then precisely mapped by screening a library of phage-displayed peptides, as located in the PA domain II between residues 412-419 thus away from the cleavage site. The authors concluded that the steric hindrance of mAb 48.3, or mAb 48.3-induced conformational changes, prevented PA_83_ cleavage. MAb 7.5G, another murine mAb, inhibited the toxicity of anthrax lethal toxin *in vitro* and *in vivo* [49]. In an assay where PA_83_ and furin were incubated for various periods of time in the presence or absence of mAb 7.5G, it was observed that there were less PA_63_ and PA_20_, and more PA_83_, in the presence of mAb 7.5G than in its absence. It was similarly concluded that mAb 7.5G inhibited PA_83_ cleavage by furin. In an ELISA, mAb 7.5G bound to the first 157 amino acids of PA_83_ domain I_,_ and thus mAb 7.5G epitope was mapped in the domain encompassing the cleavage site, well in agreement with its mechanism of action. Of note, 22G12, a potent inhibitor of anthrax LT *in vitro* and *in vivo*, bound PA_83_ near the cleavage site, as its binding was inhibited by trypsin [50] and it prevented subsequent steps necessary for toxicity. Unexpectedly, 22G12 did not prevent cleavage but was thought to inhibit the release of PA_20_, by “clipping” PA_63_ and PA_20_.

Only mAbs 7.5G and 48.3 have been positively tested *in vivo*, utilizing two murine models, intoxication with LT and subcutaneous infection with spores. Of note, the use of 48.3, in combination with mAbs 50.8 and 7.5, constituted the first oligoclonal set of mAbs that provided synergistic protection against anthrax [39]. Although a single therapeutic would be less expensive to develop, an oligoclonal mixture of mAbs may be necessary for neutralization and may also act synergistically with other antimicrobials. 

### 2.3. Antibodies Inhibiting PA Heptamerization

PA_63_ molecules, bound to their cellular receptors and separated from PA_20_, diffuse in the two dimensions of the membrane and form spontaneously heptameric complexes, which are thermodynamically favorable over the monomeric form. Mutations in the loop encompassing residues 510-518 of the PA domain III, and more particularly the residue 512, strongly inhibited this oligomerization, driving to the inhibition of the LT toxicity. These results indicated that the PA domain III was particularly involved in PA heptamerization [11,51]. 

Murine mAbs 2D3, 2D5 and 10D2 neutralized LT *in vitro*, binding to the region located between residues 581-601. Their mechanism of action was initially hypothesized to be either inhibition of PA binding to ATRs or inhibition of PA heptamerization, but the latter may be regarded as the most probable due to their epitope location [52]. The human mAb AVP-21D9 strongly neutralized *B. anthracis* and bound to PA_47_, a PA fragment corresponding to a portion of domain III, and to domain IV [53]. This mAb was shown to inhibit heptamer formation *in vitro*, possibly by masking the assembly interfaces [50]. A strongly protective mAb, MDX-1303, bound PA_83_ but it did not prevent PA_83_ interaction with ATRs. More precisely, it was shown to bind PA_63_, but it did not inhibit LF binding [54]. The MDX-1303 epitope has been located in PA_47_, as with AVP-21D9. From these elements, it was concluded that MDX-1303 inhibited heptamer assembly or function, and it was shown that the absence of MDX-1303 Fc region decreased its activity by a ten fold. It was thus inferred that the bridging of PA with Fc receptors (FcRs) was involved in the neutralization mechanism, which might depend on the inhibition of heptamer assembly or function. Of note, it was shown that the neutralizing activity of sera raised against PA also depended on Fc receptors, in an experiment where the blockade of FcRs with mAbs reduced neutralization. Thus, MDX-1303 may depend on an activity also present in sera raised by the anthrax vaccine. A third mAb, murine 1G3 mAb, reacted with PA_63_ exclusively in its heptameric form and its epitope has been located between residues 168-314, on domains I and II [52]. By electron microscopy, it was shown that 1G3 binding to heptamers may stitch two heptamers together, resulting in dodecamer-like supercomplexes [55] that prevented following steps from occurring.

Two Ab therapeutic candidates, able to neutralize PA heptamerization, are undergoing clinical trials. Valortim^®^ (PharmAthene Inc, Annapolis, MD), or MDX-1303, is currently in Phase I clinical trials [54]. NHPs exposed to 200 LD_50_ aerosolized Ames spores demonstrated complete survival after the administration of 1 mg/kg of this Ab, at 1-h post-exposure. Although Phase I studies of Valortim^®^ were either suspended or terminated due to an adverse event, ongoing investigations are underway to determine the cause of this reaction and this mAb remains in Phase I trials under study designations NCT00964834 and NCT00964561. A second human anti-PA IgG1, Thravixa^TM^ (previously AVP-21D9), has been produced by Emergent (Rockville, MD) and is currently in Phase I clinical trials under the study designation NCT01202694. Utilizing rabbit models, subcutaneous administration of Thravixa^TM^ (2 mg/kg) provided complete protection against 102 LD_50_ aerosolized Ames spores when given concurrently, and 66% protection at 24-h post challenge. Additionally, a synergistic effect was observed using combined treatments of Thravixa^TM^ with ciprofloxacin in a murine model. Although not yet assessed for tolerance, the potential for *in vivo* synergistic effects with antibiotics and the existence of two human mAbs in clinical trials highlight inhibition of PA heptamerization as a promising mechanism of protection against anthrax. 

### 2.4. Antibodies Inhibiting PA-LF/EF Interactions

After heptamer formation on the host cell surface, three LF and/or EF proteins bind to PA_63_ competitively and with high affinity (*K*_D_ = 1 nM) [56] by their *N*-terminal domains (LF_N_ and EF_N_) also called PA-binding domains (260 first residues) [6,51,57,58]. LF/EF residues involved in the PA binding site were localized by mutagenesis studies [59,63] and by the analysis of a LF-PA co-crystal structure [57]. Two regions of LF are involved in that binding. The first region is the first alpha helix (LFα1 helix), located between residues 29-50, which dock into a deep amphipathic cleft on the surface of the PA_63_ heptamer. The second region is composed of LF_N_ hydrophobic residues of the alpha helix 10 (LFα10 helix), residues L188, Y223, H229, V232, L235 and Y236, which are closely involved in an interaction with a hydrophobic region on PA, involving PA residues F202, P205, I207 and I210.

By screening a naïve phage-displayed library against cell-bound PA_63,_ three human single-chain variable fragments (scFvs) that possess neutralizing activity against anthrax toxin have been isolated and converted into Fabs, SS87, SS70 and SS73. Of these three Fabs, SS87 provided the best *in vitro* neutralization when added after PA heptamerization on the cell surface, and before the addition of LF [64]. Consequently, SS87 could not act by inhibition of PA cleavage nor heptamerization, but by inhibition of a later step which could only be the interaction between PA and EF/LF. By immunofluorescent microscopy experiments it was shown that SS87 recognized and bound to the heptamer and consequently competed with the LF/EF interaction, which was thus directly identified as the inhibition mechanism. Unfortunately, the SS87 epitope was not mapped. This mechanism of neutralization is certainly shared by the human Fab A8 [40]. This Fab was isolated from a phage displayed human scFv library and protected RAW264.7 macrophage cells against an LT challenge, similar to the cell assay described above, but no direct proof of the neutralization mechanism was given.

Apart from these anti-PA Abs, the interaction between PA and LF/EF can also be inhibited by Abs targeting LF and EF. This was first confirmed by studies using a DNA vaccine encoding LF, which established *in vivo* the protective efficacy of anti-LF Abs [65]. In 2003, the murine mAb LF8 was shown to neutralize LT *in vitro*, and to protect *in vivo* utilizing a murine model of the disease [66]. Using an electro-mobility shifting assay (EMSA), it was shown that mAb LF8 inhibited LF binding to PA, forming stable LF8-LF complexes instead of PA-LF complexes. The LF8 epitope was recently localized by a standard solid-phase peptide assay in the LF domain III, between residues 314-323, thus not in the region known to be involved in the PA-LF interaction [67]. Domain III is known to be involved in LF enzymatic activity but not in PA binding, thus this mapping was not predicted. The neutralizing mAb 10G3 possessed the same neutralization mechanism as LF8 and its epitope was mapped between residues 350-361, close to the LF8 epitope, using the same peptide assay [67,68]. This epitope was also targeted by the neutralizing mAbs 5B13B1 and 3C16C3, whose mechanism of action remained undetermined but should be identical, based on epitope mapping [69]. Domain III is located at the opposite face on LF with regard to the PA binding site (LF_N_) and thus the binding of these Abs may not inhibit the PA/LF interaction by steric hindrance, but rather by altering the conformation of LF. A highly neutralizing anti-LF scFv, 2LF, has been isolated from a phage-displayed library, built from an immunized NHP. 2LF inhibited the toxin complex formation as described for LF8 [70] and was shown to cross-react with EF. After epitope mapping with peptides failed, alanine shaving and scanning indicated that the 2LF epitope was localized in the LFα10 helix region of LF_N_, directly consistent with its neutralization mechanism (submitted manuscript). A human anti-LF IgG1, IQNLF, neutralized LT *in vitro* with an efficacy three-fold higher than the anti-PA mAb IQNPA. It conferred passive protection in A/J mice infected by the *B. anthracis* Sterne spores [31]. The IQNLF epitope was localized within the LF_N_ domain using isolated LF domains and western blot analysis, but it was not more precisely mapped. Nevertheless, the authors expected that it may block the LT formation by disrupting or preventing the interaction of LF with the PA heptamer. Recently, the mAb H10 has been isolated from a mouse immunized with LF_N_ domain [71]. As with 2LF, this mAb cross-reacted with LF and EF, due to similarities between LF_N_ and EF_N_, and neutralized both toxins *in vivo*. Regarding anti-EF Abs, the mAb 9F5 inhibited the binding of radiolabeled EF to cell-bound PA [72]. Three EF fragments were obtained by acid hydrolysis with molecular weights of 18 kDa (*N*-terminal, residues 1-156), 53 kDa (central) and 17 kDa (*C*-terminal). It was shown by immunoblot that 9F5 bound specifically the *N*-terminal fragment, and it was thus expected that 9F5 inhibited the binding of EF to the PA_63_ heptamer by targeting this first half of EF_N_, but its epitope was not more precisely mapped. 

Although no mAbs inhibiting the interaction of PA-LF/EF have entered clinical trials, the development of human mAbs such as IQNLF, SS87, Fab A8, 2LF as well as the cross-reactivity of mAb H10 demonstrated the therapeutic potential of this mechanism. Of these Abs, only IQNLF and 2LF have been tested *in vivo* utilizing murine and rat models of intoxication. Future aerosol testing utilizing rabbit and/or NHP models will help determine if Abs inhibiting PA-LF/EF interactions will have clinical applications.

### 2.5. Antibodies Inhibiting Endocytosis and Translocation

The formation of the PA_63_ heptamer stimulates the endocytosis of PA-LF/EF complexes [73]. Under the acidic conditions of the endosomes, the heptamer is driven to membrane insertion and forms a pore, protecting the toxins from lysosomal proteases and facilitating EF and LF translocation into the cytoplasm [74]. 

Two chimeric chimpanzee/human mAbs, LF10E and LF11H, bound to LF_N_ (known to be involved in the interaction with PA) but it was shown by western blot analysis that these mAbs did not inhibit the association of LF to activated PA [75]. *In vitro* studies demonstrated that these mAbs did not prevent LT formation; however, both of these mAbs inhibited the LF-driven cleavage of mitogen-activate protein kinase in the cytosol. The authors have hypothesized that, because LF10E and LF11H did not bind the enzymatic site, they inhibited the LF translocation. In addition to the inhibition of LF by this mechanism, the chimeric chimpanzee/human mAb EF13D, which bound to domain IV of EF, was shown to inhibit EF activity through the inhibition of EF binding with calmodulin (CaM) [76]. *In vitro* measurements utilizing migration under native conditions and analytical ultracentrifugation (AUC) showed that EF13D binding to EF displaced CaM bound to EF. In effect, utilizing both methods, incubation of the three proteins (EF, CaM and Fab EF13D) always resulted in a mixture of EF-Fab EF13D complexes plus free CaM. Further AUC testing utilizing CaM-EF complexes mixed with free Fab EF13D resulted in a shift to EF-Fab complexes plus free CaM. However, the authors of this study did not expected the Fab EF13D to enter the CaM rich cytoplasm, thus they suggested that the mAb EF13D protected by another mechanism. This mechanism would be the blocking of EF endocytosis or translocation through the PA heptamer pore. Although this hypothesis has yet to be confirmed, additional mechanisms may also be at play according to these authors. 

Chimpanzee/human chimeric mAbs should be well tolerated for human use, given similarities between both species. *In vivo* analysis has only been performed in murine and rat models, utilizing intravenous and footpad intoxication models [76]. Meeting the FDA animal rule would require the efficacy of these mAbs to be demonstrated in rabbits or NHPs challenged by aerosolized spores. Nevertheless, these humanized mAbs constitute the closest therapeutic products utilizing this neutralization mechanism.

## 3. Antibodies Directed Against *Bacillus anthracis* Capsule

*B. anthracis* virulence factors are not only its toxins, but also consist of a poly-γ-D-glutamic acid (γDPGA) anti-phagocytic capsule, encoded on the pXO2 plasmid. This capsule is essential for *in vivo* distribution of the bacteria from the lungs [77,78]. Although previously described as poorly immunogenic [79], the γDPGA capsule has recently become a target for the generation of both vaccine and therapeutic Ab candidates. 

The murine IgG3, mAb F26G3, was developed by immunizing mice against γDPGA [80]. When injected, this mAb elicited 90% protection in a murine pulmonary challenge model. Kozel *et al.* developed several additional murine mAbs against γDPGA, which bound to separate areas of the capsular architecture, suggesting a certain level of complexity in the structure of the capsule [81]. Chen *et al.* developed two chimeric chimpanzee/human mAbs, 11D and 4C, from chimpanzees immunized against γDPGA. The variable regions of 11D and 4C were fused to Fc portions of either γ1 or γ3 isotype [82]. The authors observed similar binding affinities with IgG1 and IgG3 versions of these Abs, but increased protection utilizing the IgG1 isotype. *In vivo* testing against an intratracheal Ames spore challenge (1,5.10^4^ spores, *i.e.*, 15 LD_50_) in a murine model elicited complete protection with a 1 mg injection given at 18-hrs pre-challenge, or as late as 20-h post-challenge, utilizing the IgG1 isotype. 

The protection elicited by 11D and 4C against an intratracheal challenge of Ames spores in murine models emphasized the advancing therapeutic development of mAbs utilizing this mechanism of protection. However, further testing utilizing rabbit and/or NHP animal models, which would fulfil the FDA animal rule, will ultimately demonstrate if these mAbs will have the protective capacity for further clinical development. 

## 4. The Particular Case of Antibody Enhancing Toxin Activity

Antibodies can paradoxically increase the cytotoxic effects of LT. Previously, antibody-dependent enhancement (ADE) of pathogenicity was only shown with mAbs developed against viruses [83]. Regarding anthrax, Mohamed *et al.* demonstrated the enhancement of LT cytotoxicity with a panel of mAbs directed against PA [84]. *In vitro* studies showed that these enhancing mAbs interacted with murine macrophage Fcγ receptors, and were hypothesized to stabilize PA on the cell surface, thus increasing the quantity of PA internalized by macrophages [84]. Recently, ADE by mAbs directed against PA was further demonstrated *in vivo* [85]. *In vitro* analysis of 17 enhancing mAbs yielded eight separate antigenic regions on PA. Of note, utilizing sub-lethal LT concentrations *in vivo*, Little *et al*. was able to induce a lethal response in Fisher rats utilizing four mAbs separately. The authors of this second study remarked that the Fcγ receptor-dependent mechanism of enhancement may not be the only one responsible for the increased LT pathogenicity. 

These results could imply that immunization with PA may elicit a response that would include such enhancing Abs, but the existence of this response should be further studied. The existence of these enhancing mAbs could explain why PA-derived vaccines are not always completely protective. This suggests the need for caution when developing Abs directed against a single antigen of anthrax for clinical purposes, and Abs neutralizing alternative antigens (e.g., LF and EF) might be needed. 

## 5. Discussion and Conclusion

*B. anthracis* is one of the biological agents most at risk of being weaponized, and it was the subject of ongoing fundamental studies even before the intentional dissemination of spores in the US, in 2001 [1]. This very sad event led to several observations, including the fact that the therapeutic window was too narrow for optimal therapy [24]. Recombinant Abs against anthrax were then actively developed because Abs of animal origin had previously been shown to increase that window, in animal models of the disease, and also shortened the duration of antibiotherapy. Those animal Abs were directed against anthrax toxins, which were also targeted by recombinant Abs, isolated at a time when the structures of these toxins have been resolved. The study of these new Abs has increased the knowledge of anthrax toxins neutralization, which now may be regarded as a model. It shows the variety of mechanisms which may be inhibited for toxin neutralization: protein-protein interactions, cleavage, heptamerization, endocytosis and translocation. Of note, the existence of certain mechanisms has been directly proven *in vitro*, such as the inhibition of interaction by EMSA experiments [66,86], while other mechanisms were hypothesized only, as a consequence of the exclusion of others. Indeed, one may be cautious with hypothesized mechanisms, particularly if others have been demonstrated and in fact resemble mechanisms recently observed elsewhere, such as neutralization of botulinum toxin proteolytic activity [87]. The inhibition of endocytosis and translocation mentioned above may thus benefit from further studies. Regarding the better established mechanisms of neutralization, it is striking that the two main Abs characteristics described long ago as essential, affinity and specificity, are indeed key. Affinity is essential because the Abs compete with a natural ligand-whether receptor, substrate, another sub-unit-to inhibit interaction, cleavage, heptamerization respectively. Nanomolar affinities are often requested for such an efficient competition. This competition also explains that Ab concentration plays a role in toxin neutralization, as it relies on a dynamic equilibrium. Specificity is the second key Abs characteristic because, in most studies, highly neutralizing Abs are shown to target exactly the region involved in the inhibited process (see Figure 3) [35,36,37,38]. However, certain studies have presented epitopes which were not located in the regions known to be involved in the inhibited process [66,67], and steric hindrance or alteration of the antigen conformation may in effect explain these results. In certain studies however, the epitopes have been localized with peptides, and it is the experience of the authors of the present review that such studies might give misleading results if they are not rigorously interpreted. In particular, the peptide thought to represent the epitope should be demonstrated as efficiently competing with the natural ligand for Ab binding. In the absence of such competing effect, an epitope mapping was re-performed by alanine scanning and results found to be completely different from those obtained with peptides (manuscript in preparation). If specificity is generally a favorable characteristic, allowing high efficiency and good tolerance, it may also represent Abs Achille’s heel because a single mutation might abolish the binding of a specific Ab if localized in its epitope. While such a mutation is difficult to envision in epitopic regions essential for protein function, this risk can be limited by the development of oligoclonal Abs [13,88]. Recombinant Abs against anthrax did not always neutralize toxins, but also targeted spores. In that case, the activation of immune effectors became key, as shown by isotype importance and the role of affinity and specificity was then certainly far less important than it was for toxins neutralization. 

The fact that Abs may enhance pathogenesis is very well exemplified in the paradigm of anthrax, and should be kept in mind when Abs target a pathogen whose host cells bear FcRs [84]. In that case in effect, IgGs interacting with the pathogen may bind to the FcRs and bring the pathogen closer to its host. If the pathogen is not neutralized, this Fc-FcRs binding is likely to increase pathogenicity. When developing Abs for therapeutic or prophylactic purposes, ADE risk may be reduced by changing Fc isotype to reduce FcRs binding, or even suppressed by replacing the Fc portion by another molecule increasing Ab fragments half-life, such as PEG. This risk is far more difficult to limit when developing vaccines.

The paradigm of anthrax demonstates that, particularly if infective pathogenesis is elucidated, such as with the central role of toxins in anthrax, Abs may be remarkable anti-infective new molecules. Such new molecules are in high demand at present, due to mounting antibioresistance [26,27]. The historical anti-infective Abs should be actively developed for prophylactic or therapeutic purposes as they were against anthrax, but in their recombinant form. 

## References

[1] Inglesby T.V., O’Toole T., Henderson D.A., Bartlett J.G., Ascher M.S., Eitzen E., Friedlander A.M., Gerberding J., Hauer J., Hughes J. (2002). Anthrax as a biological weapon, 2002: Updated recommendations for management. J. Am. Med. Assoc..

[2] Green B.D., Battisti L., Koehler T.M., Thorne C.B., Ivins B.E. (1985). Demonstration of a capsule plasmid in *Bacillus anthracis*. Infect. Immun..

[3] Friedlander A.M. (1986). Macrophages are sensitive to anthrax lethal toxin through an acid-dependent process. J. Biol. Chem..

[4] Barth H., Aktories K., Popoff M.R., Stiles B.G. (2004). Binary bacterial toxins: Biochemistry, biology, and applications of common clostridium and bacillus protein. Microbiol. Mol. Biol. Rev..

[5] Collier R.J., Young J.A. (2003). Anthrax toxin. Annu. Rev. Cell Dev. Biol..

[6] Petosa C., Collier R.J., Klimpel K.R., Leppla S.H., Liddington R.C. (1997). Crystal structure of the anthrax toxin protective antigen. Nature.

[7] Pannifer A.D., Wong T.Y., Schwarzenbacher R., Renatus M., Petosa C., Bienkowska J., Lacy D.B., Collier R.J., Park S., Leppla S.H. (2001). Crystal structure of the anthrax lethal factor. Nature.

[8] Drum C.L., Yan S.Z., Bard J., Shen Y.Q., Lu D., Soelaiman S., Grabarek Z., Bohm A., Tang W.J. (2002). Structural basis for the activation of anthrax adenylyl cyclase exotoxin by calmodulin. Nature.

[9] Lacy D.B., Wigelsworth D.J., Melnyk R.A., Harrison S.C., Collier R.J. (2004). Structure of heptameric protective antigen bound to an anthrax toxin receptor: A role for receptor in pH-dependent pore formation. Proc. Natl. Acad. Sci. USA.

[10] Santelli E., Bankston L.A., Leppla S.H., Liddington R.C. (2004). Crystal structure of a complex between anthrax toxin and its host cell receptor. Nature.

[11] Mogridge J., Mourez M., Collier R.J. (2001). Involvement of domain 3 in oligomerization by the protective antigen moiety of anthrax toxin. J. Bacteriol..

[12] Little S.F., Lowe J.R. (1991). Location of receptor-binding region of protective antigen from *Bacillus anthracis*. Biochem. Biophys. Res. Commun..

[13] Rosovitz M.J., Schuck P., Varughese M., Chopra A.P., Mehra V., Singh Y., McGinnis L.M., Leppla S.H. (2003). Alanine-scanning mutations in domain 4 of anthrax toxin protective antigen reveal residues important for binding to the cellular receptor and to a neutralizing monoclonal antibody. J. Biol. Chem..

[14] Bradley K.A., Mogridge J., Mourez M., Collier R.J., Young J.A. (2001). Identification of the cellular receptor for anthrax toxin. Nature.

[15] Scobie H.M., Rainey G.J., Bradley K.A., Young J.A. (2003). Human capillary morphogenesis protein 2 functions as an anthrax toxin receptor. Proc. Natl. Acad. Sci. USA.

[16] Vitale G., Bernardi L., Napolitani G., Mock M., Montecucco C. (2000). Susceptibility of mitogen-activated protein kinase kinase family members to proteolysis by anthrax lethal factor. Biochem. J..

[17] Duesbery N.S., Webb C.P., Leppla S.H., Gordon V.M., Klimpel K.R., Copeland T.D., Ahn N.G., Oskarsson M.K., Fukasawa K., Paull K.D. (1998). Proteolytic inactivation of map-kinase-kinase by anthrax lethal factor. Science.

[18] Leppla S.H. (1982). Anthrax toxin edema factor: A bacterial adenylate cyclase that increases cyclic amp concentrations of eukaryotic cells. Proc. Natl. Acad. Sci. USA.

[19] Leppla S.H., Arora N., Varughese M. (1999). Anthrax toxin fusion proteins for intracellular delivery of macromolecules. J. Appl. Microbiol..

[20] Quinn C.P., Singh Y., Klimpel K.R., Leppla S.H. (1991). Functional mapping of anthrax toxin lethal factor by in-frame insertion mutagenesis. J. Biol. Chem..

[21] Legge T.M. (1905). The milroy lectures on industrial anthrax: Delivered before the royal college of physicians of london. Br. Med. J..

[22] Meyerhoff A., Albrecht R., Meyer J.M., Dionne P., Higgins K., Murphy D. (2004). US food and drug administration approval of ciprofloxacin hydrochloride for management of postexposure inhalational anthrax. Clin. Infect. Dis..

[23] FDA (2001). Prescription drug product: Doxycycline and penicillin g procaine administration for inhalational anthrax (post-exposure). Fed. Regist..

[24] Shepard C.W., Soriano-Gabarro M., Zell E.R., Hayslett J., Lukacs S., Goldstein S., Factor S., Jones J., Ridzon R., Williams I. (2002). ntimicrobial postexposure prophylaxis for anthrax: Adverse events and adherence.. Emerg. Infect. Dis..

[25] CDC (2001). Update: Investigation of bioterrorism-related anthrax and adverse events from antimicrobial prophylaxis.DOI: PubMed:. MMWR Morb. Mortal. Wkly. Rep..

[26] Beharry Z., Chen H., Gadhachanda V.R., Buynak J.D., Palzkill T. (2004). Evaluation of penicillin-based inhibitors of the class a and b beta-lactamases from *Bacillus anthracis*. Biochem. Biophys. Res. Commun..

[27] Chen Y., Tenover F.C., Koehler T.M. (2004). Beta-lactamase gene expression in a penicillin-resistant *Bacillus anthracis* strain. Antimicrob. Agents Chemother..

[28] Athamna A., Athamna M., Abu-Rashed N., Medlej B., Bast D.J., Rubinstein E. (2004). Selection of *Bacillus anthracis* isolates resistant to antibiotics. J. Antimicrob. Chemother..

[29] Price L.B., Vogler A., Pearson T., Busch J.D., Schupp J.M., Keim P. (2003). In vitro selection and characterization of *Bacillus anthracis* mutants with high-level resistance to ciprofloxacin. Antimicrob. Agents Chemother..

[30] Choe C.H., Bouhaouala S.S., Brook I., Elliot T.B., Knudson G.B. (2000). *In vitro* development of resistance to ofloxacin and doxycycline in *Bacillus anthracis* sterne. Antimicrob.DOI: PubMed:. Agents Chemother..

[31] Albrecht M.T., Li H., Williamson E.D., LeButt C.S., Flick-Smith H.C., Quinn C.P., Westra H., Galloway D., Mateczun A., Goldman S. (2007). Human monoclonal antibodies against anthrax lethal factor and protective antigen act independently to protect against *Bacillus anthracis* infection and enhance endogenous immunity to anthrax. Infect. Immun..

[32] Wigelsworth D.J., Krantz B.A., Christensen K.A., Lacy D.B., Juris S.J., Collier R.J. (2004). Binding stoichiometry and kinetics of the interaction of a human anthrax toxin receptor, cmg2, with protective antige. J. Biol. Chem..

[33] Lacy D.B., Wigelsworth D.J., Scobie H.M., Young J.A., Collier R.J. (2004). Crystal structure of the von willebrand factor a domain of human capillary morphogenesis protein 2: An anthrax toxin receptor. Proc. Natl. Acad. Sci. USA.

[34] Bradley K.A., Mogridge J., Jonah G., Rainey A., Batty S., Young J.A. (2003). Binding of anthrax toxin to its receptor is similar to alpha integrin-ligand interactions. J. Biol. Chem..

[35] Little S.F., Leppla S.H., Cora E. (1988). Production and characterization of monoclonal antibodies to the protective antigen component of *Bacillus anthracis* toxin. Infect. Immun..

[36] Laffly E., Danjou L., Condemine F., Vidal D., Drouet E., Lefranc M.P., Bottex C., Thullier P. (2005). Selection of a macaque fab with framework regions like those in humans, high affinity, and ability to neutralize the protective antigen (pa) of *Bacillus anthracis* by binding to the segment of pa between residues 686 and 69. Antimicrob. Agents Chemother..

[37] Kelly-Cirino C.D., Mantis N.J. (2009). Neutralizing monoclonal antibodies directed against defined linear epitopes on domain 4 of anthrax protective antigen. Infect. Immun..

[38] Chen Z., Moayeri M., Zhou Y.H., Leppla S., Emerson S., Sebrell A., Yu F., Svitel J., Schuck P., St Claire M. (2006). Efficient neutralization of anthrax toxin by chimpanzee monoclonal antibodies against protective antigen. J. Infect. Dis..

[39] Brossier F., Levy M., Landier A., Lafaye P., Mock M. (2004). Functional analysis of *Bacillus anthracis* protective antigen by using neutralizing monoclonal antibodies. Infect. Immun..

[40] Zhou B., Carney C., Janda K.D. (2008). Selection and characterization of human antibodies neutralizing *Bacillus anthracis* toxin. Bioorg. Med. Chem..

[41] Mazumdar S. (2009). Raxibacumab. MAbs.

[42] Wild M.A., Xin H., Maruyama T., Nolan M.J., Calveley P.M., Malone J.D., Wallace M.R., Bowdish K.S. (2003). Human antibodies from immunized donors are protective against anthrax toxin *in vivo*.DOI: PubMed:. Nat, Biotechnol..

[43] Migone T.S., Subramanian G.M., Zhong J., Healey L.M., Corey A., Devalaraja M., Lo L., Ullrich S., Zimmerman J., Chen A. (2009). Raxibacumab for the treatment of inhalational anthrax. N. Engl. J. Med..

[44] Antoniu S.A. (2010). Raxibacumab for inhalational anthrax: An effective specific therapeutic approach? Expert Opin. Investig. Drugs.

[45] Mohamed N., Clagett M., Li J., Jones S., Pincus S., D’Alia G., Nardone L., Babin M., Spitalny G., Casey L. (2005). A high-affinity monoclonal antibody to anthrax protective antigen passively protects rabbits before and after aerosolized *Bacillus anthracis* spore challenge. Infect. Immun..

[46] Klimpel K.R., Molloy S.S., Thomas G., Leppla S.H. (1992). Anthrax toxin protective antigen is activated by a cell surface protease with the sequence specificity and catalytic properties of furin. Proc. Natl. Acad. Sci. USA.

[47] Singh Y., Chaudhary V.K., Leppla S.H. (1989). A deleted variant of *Bacillus anthracis* protective antigen is non-toxic and blocks anthrax toxin action *in vivo*. J. Biol. Chem..

[48] Christensen K.A., Krantz B.A., Melnyk R.A., Collier R.J. (2005). Interaction of the 20 kda and 63 kda fragments of anthrax protective antigen: Kinetics and thermodynamics. Biochemistry.

[49] Rivera J., Nakouzi A., Abboud N., Revskaya E., Goldman D., Collier R.J., Dadachova E., Casadevall A. (2006). A monoclonal antibody to *Bacillus anthracis* protective antigen defines a neutralizing epitope in domain 1. Infect. Immun..

[50] Wang F., Ruther P., Jiang I., Sawada-Hirai R., Sun S.M., Nedellec R., Morrow P.R., Kang A.S. (2004). Human monoclonal antibodies that neutralize anthrax toxin by inhibiting heptamer assembly. Hum. Antib..

[51] Milne J.C., Furlong D., Hanna P.C., Wall J.S., Collier R.J. (1994). Anthrax protective antigen forms oligomers during intoxication of mammalian cells. J. Biol. Chem..

[52] Little S.F., Novak J.M., Lowe J.R., Leppla S.H., Singh Y., Klimpel K.R., Lidgerding B.C., Friedlander A.M. (1996). Characterization of lethal factor binding and cell receptor binding domains of protective antigen of *Bacillus anthracis* using monoclonal antibodies. Microbiology.

[53] Peterson J.W., Comer J.E., Baze W.B., Noffsinger D.M., Wenglikowski A., Walberg K.G., Hardcastle J., Pawlik J., Bush K., Taormina J. (2007). Human monoclonal antibody avp-21d9 to protective antigen reduces dissemination of the *Bacillus anthracis* ames strain from the lungs in a rabbit model.. Infect. Immun..

[54] Vitale L., Blanset D., Lowy I., O’Neill T., Goldstein J., Little S.F., Andrews G.P., Dorough G., Taylor R.K., Keler T. (2006). Prophylaxis and therapy of inhalational anthrax by a novel monoclonal antibody to protective antigen that mimics vaccine-induced immunity. Infect. Immun..

[55] Radjainia M., Hyun J.K., Leysath C.E., Leppla S.H., Mitra A.K. (2010). Anthrax toxin-neutralizing antibody reconfigures the protective antigen heptamer into a supercomplex. Proc. Natl. Acad. Sci. USA.

[56] Elliott J.L., Mogridge J., Collier R.J. (2000). A quantitative study of the interactions of *Bacillus anthracis* edema factor and lethal factor with activated protective antigen. Biochemistry.

[57] Feld G.K., Thoren K.L., Kintzer A.F., Sterling H.J., Tang I.I., Greenberg S.G., Williams E.R., Krantz B.A. (2010). Structural basis for the unfolding of anthrax lethal factor by protective antigen oligomers. Nat. Struct. Mol. Biol..

[58] Kintzer A.F., Thoren K.L., Sterling H.J., Dong K.C., Feld G.K., Tang I.I., Zhang T.T., Williams E.R., Berger J.M., Krantz B.A. (2009). The protective antigen component of anthrax toxin forms functional octameric complexes. J. Mol. Biol..

[59] Cunningham K., Lacy D.B., Mogridge J., Collier R.J. (2002). Mapping the lethal factor and edema factor binding sites on oligomeric anthrax protective antigen. Proc. Natl. Acad. Sci. USA.

[60] Kumar P., Ahuja N., Bhatnagar R. (2001). Purification of anthrax edema factor from *Escherichia coli* and identification of residues required for binding to anthrax protective antigen. Infect. Immun..

[61] Lacy D.B., Lin H.C., Melnyk R.A., Schueler-Furman O., Reither L., Cunningham K., Baker D., Collier R.J. (2005). A model of anthrax toxin lethal factor bound to protective antigen. Proc. Natl. Acad. Sci. USA.

[62] Lacy D.B., Mourez M., Fouassier A., Collier R.J. (2002). Mapping the anthrax protective antigen binding site on the lethal and edema factors. J. Biol. Chem..

[63] Melnyk R.A., Hewitt K.M., Lacy D.B., Lin H.C., Gessner C.R., Li S., Woods V.L., Collier R.J. (2006). Structural determinants for the binding of anthrax lethal factor to oligomeric protective antigen. J. Biol. Chem..

[64] Steiniger S.C., Altobell L.J., Zhou B., Janda K.D. (2007). Selection of human antibodies against cell surface-associated oligomeric anthrax protective antigen. Mol. Immunol..

[65] Price B.M., Liner A.L., Park S., Leppla S.H., Mateczun A., Galloway D.R. (2001). Protection against anthrax lethal toxin challenge by genetic immunization with a plasmid encoding the lethal factor protein. Infect. Immun..

[66] Zhao P., Liang X., Kalbfleisch J., Koo H.M., Cao B. (2003). Neutralizing monoclonal antibody against anthrax lethal factor inhibits intoxication in a mouse model. Hum. Antib..

[67] Nguyen M.L., Crowe S.R., Kurella S., Teryzan S., Cao B., Ballard J.D., James J.A., Farris A.D. (2009). Sequential b-cell epitopes of *Bacillus anthracis* lethal factor bind lethal toxin-neutralizing antibodies. Infect. Immun..

[68] Little S.F., Leppla S.H., Friedlander A.M. (1990). Production and characterization of monoclonal antibodies against the lethal factor component of *Bacillus anthracis* lethal toxin. Infect. Immun..

[69] Lim N.K., Kim J.H., Oh M.S., Lee S., Kim S.Y., Kim K.S., Kang H.J., Hong H.J., Inn K.S. (2005). An anthrax lethal factor-neutralizing monoclonal antibody protects rats before and after challenge with anthrax toxin. Infect. Immun..

[70] Pelat T., Hust M., Laffly E., Condemine F., Bottex C., Vidal D., Lefranc M.P., Dubel S., Thullier P. (2007). High-affinity, human antibody-like antibody fragment (single-chain variable fragment) neutralizing the lethal factor (lf) of *Bacillus anthracis* by inhibiting protective antigen-lf complex formation. Antimicrob. Agents Chemother..

[71] Kulshreshtha P., Bhatnagar R. (2011). Inhibition of anthrax toxins with a bispecific monoclonal antibody that cross reacts with edema factor as well as lethal factor of *Bacillus anthracis*. Mol. Immunol..

[72] Little S.F., Leppla S.H., Burnett J.W., Friedlander A.M. (1994). Structure-function analysis of *Bacillus anthracis* edema factor by using monoclonal antibodies. Biochem. Biophys. Res. Commun..

[73] Abrami L., Liu S., Cosson P., Leppla S.H., van der Goot F.G. (2003). Anthrax toxin triggers endocytosis of its receptor via a lipid raft-mediated clathrin-dependent process. J. Cell Biol..

[74] Abrami L., Lindsay M., Parton R.G., Leppla S.H., van der Goot F.G. (2004). Membrane insertion of anthrax protective antigen and cytoplasmic delivery of lethal factor occur at different stages of the endocytic pathway. J. Cell Biol..

[75] Chen Z., Moayeri M., Crown D., Emerson S., Gorshkova I., Schuck P., Leppla S.H., Purcell R.H. (2009). Novel chimpanzee/human monoclonal antibodies that neutralize anthrax lethal factor, and evidence for possible synergy with anti-protective antigen antibody. Infect. Immun..

[76] Chen Z., Moayeri M., Zhao H., Crown D., Leppla S.H., Purcell R.H. (2009). Potent neutralization of anthrax edema toxin by a humanized monoclonal antibody that competes with calmodulin for edema factor binding. Proc. Natl. Acad. Sci. USA.

[77] Ivins B.E., Welkos S.L. (1986). Cloning and expression of the *Bacillus anthracis* protective antigen gene in bacillus subtilis. Infect. Immun..

[78] Drysdale M., Heninger S., Hutt J., Chen Y., Lyons C.R., Koehler T.M. (2005). Capsule synthesis by *Bacillus anthracis* is required for dissemination in murine inhalation anthrax. Embo J..

[79] Wang T.T., Fellows P.F., Leighton T.J., Lucas A.H. (2004). Induction of opsonic antibodies to the gamma-d-glutamic acid capsule of *Bacillus anthracis* by immunization with a synthetic peptide-carrier protein conjugate. FEMS Immunol. Med. Microbiol..

[80] Kozel T.R., Murphy W.J., Brandt S., Blazar B.R., Lovchik J.A., Thorkildson P., Percival A., Lyons C.R. (2004). Mabs to *Bacillus anthracis* capsular antigen for immunoprotection in anthrax and detection of antigenemia. Proc. Natl. Acad. Sci. USA.

[81] Kozel T.R., Thorkildson P., Brandt S., Welch W.H., Lovchik J.A., AuCoin D.P., Vilai J., Lyons C.R. (2007). Protective and immunochemical activities of monoclonal antibodies reactive with the *Bacillus anthracis* polypeptide capsule. Infect. Immun..

[82] Chen Z., Schneerson R., Lovchik J., Lyons C.R., Zhao H., Dai Z., Kubler-Kielb J., Leppla S.H., Purcell R.H. (2011). Pre- and postexposure protection against virulent anthrax infection in mice by humanized monoclonal antibodies to *Bacillus anthracis* capsule. Proc. Natl. Acad. Sci. USA.

[83] Tirado S.M., Yoon K.J. (2003). Antibody-dependent enhancement of virus infection and disease. Viral Immunol..

[84] Mohamed N., Li J., Ferreira C.S., Little S.F., Friedlander A.M., Spitalny G.L., Casey L.S. (2004). Enhancement of anthrax lethal toxin cytotoxicity: A subset of monoclonal antibodies against protective antigen increases lethal toxin-mediated killing of murine macrophages. Infect. Immun..

[85] Little S.F., Webster W.M., Fisher D.E. (2011). Monoclonal antibodies directed against protective antigen of *Bacillus anthracis* enhance lethal toxin activity *in vivo*. FEMS Immunol. Med. Microbiol..

[86] Pelat T., Hüst M., Laffly E., Condemine F., Bottex C., Vidal D., Lefranc M.P., Dübel S., Thullier P. (2007). High-affinity, human antibody-like antibody fragment (single-chain variable fragment) neutralizing the lethal factor (lf) of *Bacillus anthracis* by inhibiting protective antigen-lf complex formation. Antimicrob. Agents Chemother..

[87] Adekar S.P., Takahashi T., Jones R.M., Al-Saleem F.H., Ancharski D.M., Root M.J., Kapadnis B.P., Simpson L.L., Dessain S.K. (2008). Neutralization of botulinum neurotoxin by a human monoclonal antibody specific for the catalytic light chain. PLoS One.

[88] Baillie L.W. (2006). Past, imminent and future human medical countermeasures for anthrax. J. Appl. Microbiol..

